# Crystalline Silica Impairs Efferocytosis Abilities of Human and Mouse Macrophages: Implication for Silica-Associated Systemic Sclerosis

**DOI:** 10.3389/fimmu.2020.00219

**Published:** 2020-02-18

**Authors:** Alain Lescoat, Alice Ballerie, Marie Lelong, Yu Augagneur, Claudie Morzadec, Stéphane Jouneau, Patrick Jégo, Olivier Fardel, Laurent Vernhet, Valérie Lecureur

**Affiliations:** ^1^Univ Rennes, CHU Rennes, Inserm, EHESP, Irset (Institut de Recherche en Santé, Environnement et Travail) – UMR_S 1085, Rennes, France; ^2^Department of Internal Medicine and Clinical Immunology, Rennes University Hospital, Rennes, France; ^3^Department of Respiratory Diseases, Rennes University Hospital, Rennes, France; ^4^Pôle Biologie, Rennes University Hospital, Rennes, France

**Keywords:** crystalline silica, efferocytosis, macrophage polarization, rho kinase, systemic sclerosis

## Abstract

Inhalation of crystalline silica (SiO_2_) is a risk factor of systemic autoimmune diseases such as systemic sclerosis (SSc) and fibrotic pulmonary disorders such as silicosis. A defect of apoptotic cell clearance (i.e., efferocytosis, a key process in the resolution of inflammation) is reported in macrophages from patients with fibrotic or autoimmune diseases. However, the precise links between SiO_2_ exposure and efferocytosis impairment remain to be determined. Answering to this question may help to better link innate immunity and fibrosis. In this study, we first aim to determine whether SiO_2_ might alter efferocytosis capacities of human and mouse macrophages. We secondly explore possible mechanisms explaining efferocytosis impairment, with a specific focus on macrophage polarization and on the RhoA/ROCK pathway, a key regulator of cytoskeleton remodeling and phagocytosis. Human monocyte-derived macrophages (MDM) and C57BL/6J mice exposed to SiO_2_ and to CFSE-positive apoptotic Jurkat cells were analyzed by flow cytometry to determine their efferocytosis index (EI). The effects of ROCK inhibitors (Y27632 and Fasudil) on EI of SiO_2_-exposed MDM and MDM from SSc patients were evaluated *in vitro*. Our results demonstrated that SiO_2_ significantly decreased EI of human MDM *in vitro* and mouse alveolar macrophages *in vivo*. In human MDM, this SiO_2_-associated impairment of efferocytosis, required the expression of the membrane receptor SR-B1 and was associated with a decreased expression of M2 polarization markers (CD206, CD204, and CD163). F-actin staining, RhoA activation and impairment of efferocytosis, all induced by SiO_2_, were reversed by ROCK inhibitors. Moreover, the EI of MDM from SSc patients was similar to the EI of *in vitro*- SiO_2_-exposed MDM and Y27632 significantly increased SSc MDM efferocytosis capacities, suggesting a likewise activation of the RhoA/ROCK pathway in SSc. Altogether, our results demonstrate that SiO_2_ exposure may contribute to the impairment of efferocytosis capacities of mouse and human macrophages but also of MDM in SiO_2_-associated autoimmune diseases and fibrotic disorders such as SSc; in this context, the silica/RhoA/ROCK pathway may constitute a relevant therapeutic target.

## Introduction

Exploring the pathogenesis of silica-associated fibrotic and autoimmune disorders may help to decipher the link between fibrotic diseases and autoimmunity. Indeed, the consequences of crystalline silica (SiO_2_) on health firstly include respiratory disorders, and more specifically, silicosis, a granulomatous and fibrotic interstitial lung disease (1). Beyond these direct pulmonary effects, independent epidemiological studies show that exposure to crystalline silica increases the risk of autoimmune connective tissue diseases (CTD) with chronic inflammation such as systemic sclerosis (SSc) or systemic lupus erythematosus (SLE) (2–4). SSc is considered as the main autoimmune disorder associated with silica exposure and, among rheumatic diseases, this chronic inflammatory and fibrotic disease involving lung and skin, has the highest case-specific mortality (5). Almost 50% of men suffering from SSc may have a history of silica exposure (6, 7). Crystalline silica is an oxide of silicon commonly found in nature as quartz. Exposure to crystalline silica particles especially occurs in occupational settings when materials containing crystalline silica are reduced to dust (1). More than 30% of workers could suffer from silica hazards in primary industries and high-risk sectors in developing countries (8). The pathogenic links between silica inhalation and the onset of fibrotic autoimmune disorders such as SSc are still to be determined.

Lungs and respiratory tracts constitute the first tissue interacting with SiO_2_ after inhalation. As pulmonary macrophages (MΦ) phagocyte SiO_2_ particles, they are considered the main cellular targets of this airborne contaminant (9). Via interaction with the membrane scavenger receptor B1 (SR-B1), SiO_2_ exerts pro-inflammatory effects on MΦ through activation of the NLRP3 inflammasome (10, 11). MΦ are also involved in the resolution of inflammation. They can indeed adopt various phenotypes or activation states, either pro- or anti-inflammatory (i.e., M1 or M2 MΦ, respectively), according to their surrounding microenvironment. An altered MΦ polarization has been described in SSc (12) and in SLE (13) and also in fibrotic pulmonary diseases such as idiopathic pulmonary fibrosis (IPF) (14). Nonetheless, the effects of SiO_2_ on the polarization states and associated phenotypes of MΦ are further to be explored.

Among MΦ properties, the process of efferocytosis, i.e., the specialized recognition and ingestion of apoptotic cells, is also essential for tissue homeostasis. Efferocytosis is a key process in the resolution of inflammation. By processing the clearance of apoptotic bodies, MΦ limit inflammation by preventing the secondary necrosis of apoptotic cells. Impaired efferocytosis can also participate to the release of auto-antigens, such as intra-nuclear components of apoptotic cells, which can directly contribute to the triggering of systemic autoimmunity (15–17). Interestingly, an impaired efferocytosis has been documented in monocyte-derived macrophages (MDM) from patient with systemic autoimmune disorders such as SLE (18–20) or SSc (21) but also in fibrotic diseases such as IPF (22). Efferocytosis could represent a key pathogenic process at the crossroads of systemic auto-immunity, MΦ and fibrosis. As SiO_2_ is an airborne contaminant associated with both systemic autoimmunity and pulmonary fibrosis, exploring the consequences of SiO_2_ on MΦ polarization and function may provide new insights on the link between autoimmunity, chronic inflammation and fibrosis. Nonetheless, the direct impact of SiO_2_ exposure on efferocytosis capacities and polarization of MΦ has never been studied to date. In the present study, we first aim to determine whether SiO_2_ might alter efferocytosis capacities of human and mouse MΦ. We secondly explore possible mechanisms that could explain an impaired efferocytosis, with a specific focus on MΦ polarization and on the RhoA/ROCK pathway, a key regulator of cell adhesion, cytoskeleton remodeling and phagocytosis, that has been recently advanced as a promising therapeutic target in fibrotic disease and especially in SSc (23, 24).

## Materials and Methods

### Reagents

Crystalline silica particles (SiO_2_, DQ 12; d_50_ = 2.2 μm, DMT GmbH & Co.KG, Essen, Germany) and Tungsten carbide (WC) particles (d_50_ = 1 μm) were heated at 200°C for 2 h to remove any possible endotoxin contamination, suspended in sterile water at the concentration of 100 mg/ml and were then sonicated for 30 min. Human recombinant cytokine IFNɤ, IL-4, and IL-13 were purchased from Peprotech (Neuilly sur Seine, France) and human recombinant GM-CSF and M-CSF were obtained from Sanofi-Aventis (Montrouge, France) and Miltenyi Biotec SAS (Paris, France), respectively. Camptothecin, propidium iodide (IP) and Lipopolysaccharide (LPS) from *E. coli* (serotype: 055:B5) were purchased from Sigma-Aldrich (St-Quentin Fallavier, France). FITC-Annexin V was purchased from BD Biosciences (Le Pont de Claix, France). Fasudil was obtained from MedchemExpress whereas the Rho-associated protein kinase (ROCK) inhibitor (+)-C-trans-4-(1-aminoethyl)-N- (4-pyridyl) cyclohexane carboxamide (Y27632) was purchased from Santa cruz Biotechnology, INC (Heidelberg).

### Preparation of Human Monocyte-Derived Macrophages (MDM)

#### Patients With SSc and Healthy Donors (HD)

Peripheral blood mononuclear cells were obtained from HD or SSc patients through Ficoll gradient centrifugation. SSc patients from the department of Internal Medicine and Clinical Immunology of Rennes University Hospital were consecutively included after written informed consent. All patients fulfilled the 2013 ACR/EULAR classification criteria for SSc (25). Patients with overlapping syndrome with Sjögren syndrome or SLE were not included. Blood buffy coats of healthy donors were provided by Etablissement Français du Sang (Rennes, France) after consent. All healthy donors included in this study answered a medical questionnaire; allowing the exclusion of any pathologic condition (acute or chronic).

#### Differentiation of Blood Monocytes in MDM and Treatment

In all experiments, monocytes were selected after a 1 h adhesion step and were differentiated into MΦ for 6 days using GM-CSF(400 IU/ml) or M–CSF (50 ng/ml) in RPMI 1640 medium GlutaMAX (Gibco, Life technologies SAS, Courtaboeuf, France) supplemented with 10 % heat-inactivated fetal bovine serum (FBS, Lonza, Levallois-Perret, France), 20 IU/ml penicillin and 20 μg/ml streptomycin (ThermoFisher Scientific, Courtaboeuf, France). Unless otherwise indicated, M0-MDM from HD were exposed *in vitro* to SiO_2_ as follows: particles were re-suspended by vortexing before their addition to the medium for 4 h, MDM were then washed and subsequent experiments were performed.

#### *In vitro* Polarization of MDM

For M1 polarization, MDM were activated for additional 24 h by the addition of IFNɤ (20 ng/ml) and LPS (20 ng/ml). For M2a polarization, MDM were activated for additional 24 h by the addition of IL-4 (20 ng/ml) and IL-13 (20 ng/ml). Before treatment, all MDM were placed in medium with 5% of FBS. For experiments described in **Figure 7**, M-CSF was replaced by GM-CSF (400 IU/ml) in the same conditions, to obtain GM-MDM (26).

### Cell Viability

Cytotoxic effects of SiO_2_ treatment on human MDM were assessed using reagent WST-1 colorimetric assay (Cell proliferation Reagent, Roche, Mannheim, Germany). Briefly, 4-day MDM were seeded in 96-well-plates at 0.4 × 10^5^ cells/well to achieve their differentiation. Six day-old MDM were then exposed for 24 h to various concentrations of particles. After silica exposure, cells were washed twice, and 100 μL of medium with 10% of WST-1 was added in each well. Absorbance of soluble formazan formed products was measured after 60 min and 90 min at 450 nm using SPECTROstar Nano (BMG Labtech, Ortenberg, Germany). For some experiment, MDM cell viability was also evaluated by flow cytometry through the analysis of the percentage of Annexin-V-IP staining positive cells as previously described (21).

### Animal Protocols

Female C57BL/6J mice weighing between 18 and 20 gr, used at 8 weeks of age, purchased from Janvier Labs (Le Genest Saint Isle, France) were randomly divided into 3 groups (*n* = 5 per experimental group). The animals were housed in positive pressure air-conditioned units (25°C, 50% relative humidity) on a 12-h light/dark cycle. For instillation, animals were anesthetized with a mix of ketamine and xylazine (respectively, 60 and 10 mg/kg). Particles were suspended in NaCl 0.9% and 1.5 mg of particles (SiO_2_ or WC) per mouse (50 μl/mouse) were instilled into the lungs via trachea by transoral instillation. Control mice were instilled with the corresponding volume of NaCl. Four days after particle instillation, 5 × 10^6^ CFSE^pos^ apoptotic Jurkat cells in 50 μl saline were administered into the lungs by transoral instillation. Mice were sacrificed 3 h after apoptotic cell instillation with an overdose of ketamine and xylazine (respectively, 100 and 20 mg/kg). Bronchoalveolar lavages (BAL) were performed by cannulating the trachea (with a 21G needle) and infusing the lungs five times with 1 ml of NaCl 0.9%. The BAL fluids were centrifuged (800 g, 10 min, 4°C) and cell pellets were re-suspended in PBS-2% FBS for flow cytometry analyses with determination of the efferocytosis index (EI).

### Efferocytosis Assays

Human Jurkat CD4 T-lymphocyte cells (1.10^6^ cells/ml), cultured in RPMI 1640 Glutamax culture medium with 10 % FBS, 20 IU/ml penicillin and 20 μg/ml streptomycin, were stained for 15 min with 100 ng/ml CellTrace^TM^ CFSE (Invitrogen), washed and then exposed for 4 h to 10 μM camptothecin to induce apoptosis as previously described (21).

#### Human MDM Analysis

CFSE-stained apoptotic and non-apoptotic Jurkat cells were added to MDM plated in 12-well-tissue culture plates, in 10:1 ratio (apoptotic cells/MΦ) for 90 min at 37°C in a 5% CO_2_ humidified incubator. After co-culture, Jurkat cells were removed, MΦ were washed at least 3 times with phosphate-buffered saline (PBS), detached using Accutase^TM^ (BioLegends, Paris, France) and incubated with Fc-block (Miltenyi Biotech SAS) in a PBS supplemented with 2% FBS solution. The staining of Jurkat cells with human anti-CD3-PE antibody (BD Biosciences) was used to exclude MΦ with unengulfed lymphocytes bound to their surface. Engulfment efficiency was measured by flow cytometry. EI was calculated according to the following equation: EI = (number of CD3^neg^ CFSE^pos^ MΦ /number of total MΦ) × 100.

#### BAL Fluids Analysis

As previously described, mouse cells from BAL were re-suspended in PBS supplemented with 2% FBS solution containing Fc block and then stained with human anti-CD3-PE, mouse anti-CD11b-PE-Cy7 (BD Biosciences) and anti-Gr1-V450 (eBiosciences SAS, Paris) antibodies. Engulfment efficiency was measured by flow cytometry. EI was calculated according to the following equation: EI = (number of CD3^neg^ Gr1^int^ CD11b^Int^ CFSE^pos^ cells /number of total CD3^neg^ Gr1^int^ CD11b^Int^ cells) × 100.

### Cell Surface Marker Analyses by Flow Cytometry

After cell detachment using Accutase^TM^, MDM were first blocked in PBS supplemented with 2% FBS solution and with Fc block, then re-suspended and incubated with specific antibodies or appropriate isotypic controls for 30 min at 4°C. Cells were then washed once with PBS and analyzed on a LSR II cytometer with FACSDiva software (BD Biosciences). Surface marker expression was evaluated using the following antibodies: anti-CD163-FITC, anti-CD206-PE (BD Biosciences), anti-CD204-PE (R&D Systems, Abingdon, UK), anti-CD91-vioBright FITC, anti-CD44-vioBlue, anti-SR-B1-APC, anti-integrin αV, β5, or β3 (all from Miltenyi Biotec, SAS). Results were expressed as a ratio of median fluorescence intensity (MFI) calculated as follows: median fluorescence (mAb of interest)/ median fluorescence (isotype control mAb).

### Transfection of siRNA

SMARTpool of individual siRNAs directed against human SCARB1 (SR-B1) (L-010592-00-0005) and a non-targeting pool (siRNA Ct), used as control, were purchased from Dharmacon (GE Healthcare Europe GmbH-FR, Velizy-Villacoublay, France). MDM were transfected using Lipofectamine RNAiMax reagent (Invitrogen) with siRNA at 5 pmol for 24 h. Silencing efficiency of siRNA SR-B1 was analyzed at the protein level using the anti-SR-B1-APC antibody (Miltenyi Biotec, SAS) by flow cytometry on a LSR II cytometer.

### Analysis of F-actin Expression

Four-days MDM were trypsinized with Accutase^TM^ and then plated at 18 × 10^3^ cells/cm^2^ on a Lab-Tek^TM^ chamber slide system (Thermo Fisher Scientific, France) for additional 24 h. Twenty-four hours before treatment, cell culture medium was changed and replaced by a medium with 1% of FBS. MDM were next pre-treated or not with 20 μM of Y27632 for 1 h and, they were untreated or treated with 25 μg/cm^2^ of SiO_2_ or they were polarized into M1 MDM. Cells were fixed with 4% paraformaldehyde for 20 min at room temperature, washed three times with PBS and permeabilized in a 0.2% Triton X100 for 5 min and blocked in PBS containing 4% BSA for 1 h at room temperature. MDM were then incubated with Alexa fluor 568-phalloidin (Life Technologies SAS), to detect F-actin filaments, in a blocking solution for 2 h at room temperature and then washed with PBS. Thereafter, cells were co-stained with DAPI (Thermo Fisher Scientific), a fluorescent dye specific for DNA, for 10 min. After washings, coverslips were mounted with Dako Fluorescence mounting medium (Agilent Technologies France). Fluorescent-labeled cells were captured with a fluorescence microscope (Zeiss Apotome, Axio Imager Z1) and quantification of phalloidin staining was performed with Image J 1.52a software (NIH, USA).

### Western Blotting

MDM were harvested and lysed on ice in RIPA buffer supplemented with phosphatase inhibitor cocktail 2 and 3 (Sigma-Aldrich) and a cocktail of protein inhibitors (Roche Diagnostic, Meylan, France). Then, cell lysates were sonicated on ice and protein concentration was measured using the Bradford's method. Samples were heated for 5 min at 100°C, loaded in a 4 % stacking gel and then separated by a 8% sodium dodecyl sulfate polymerase gel electrophoresis (SDS-PAGE). Gels were electroblotted overnight onto nitrocellulose membranes. After blocking the membrane with a Tris-buffered saline solution supplemented with 0.1% tween-20 and 5% bovine serum albumin, membranes were hybridized with primary antibodies overnight at 4°C and incubated with appropriate horseradish peroxidase-conjugated (HRP) secondary antibodies. Primary antibodies used were directed against anti-P-MYPT1 (Thr696) (Ozyme SAS, Saint Quentin-en-Yvelines, France) and anti-HSC70 (Santa Cruz Biotechnology, Inc. Heidelberg, Germany). Immunolabeled proteins were finally visualized by chemiluminescence. Full scans of the entire original gels are provided as supplementary material. Densitometry with ImageJ 1.40g software (National Institutes of Health, Bethesda, MD, USA) was used for quantifying intensities of stained bands and normalization to HSC70 content.

### RhoA-GTP Pull-Down Assay

Twenty-four hours before treatment, MDM culture medium was changed and replaced by a medium with 1 % of FBS. RhoA-GTP levels were measured using the RhoA activation assay biochem kit^TM^ (Cytoskeleton, Tebu-bio, Le Perray-en Yvelines, France). Briefly, cells were rapidly lysed at 4°C and equal volumes of 300 μg total cellular proteins were incubated with 50 μg of Rhotekin-RBD beads to specifically pull-down RhoA-GTP. After washing, the beads were re-suspended in Laemmli buffer, boiled, and subjected to Western-blot analysis. SDS-PAGE and Western blotting were performed as described above, by using primary anti-RhoA or anti-HSC70 antibodies.

### Statistical Analysis

Data are presented as means ± standard error on the mean (SEM). Comparison between more than 2 groups were performed by one-way analysis of variance followed by Dunnett's or Newman–Keuls multiple comparison *post-hoc* tests. Depending on conditions and Gaussian distribution, Student's *t* test, paired-*t*-test or Mann–Whitney test were used to compare 2 groups. A *P* < 0.05 was considered significant. Data analyzes were performed with GraphPad Prism 5.0 software (GraphPad Software, La Jolla, CA, USA).

## Results

### Impaired Efferocytosis Capacities of Human SiO_2_-Exposed MDM and Alveolar Macrophages of Mice Exposed to SiO_2_

**Figure 1:**
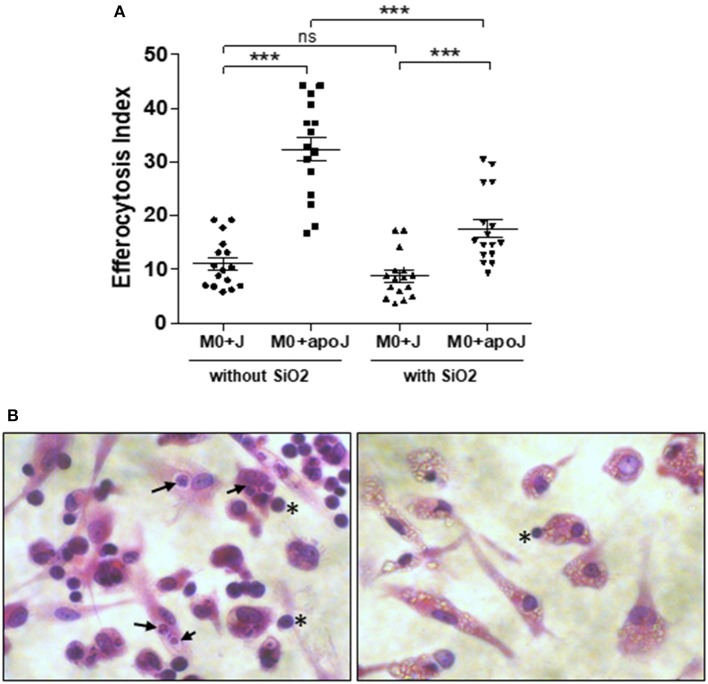
*In vitro* impaired efferocytosis capacities of silica-exposed MDM. **(A)** EI of M0 MDM from the same healthy donors untreated or treated by 25 μg/cm^2^ of SiO_2_ for 4 h and then exposed to apoptotic Jurkat (apoJ) or live Jurkat (J) cells. Efferocytosis assay was performed on MDM from 16 different healthy donors. **(B)** Pictures of optical microscopy: M0 MDM were untreated (left) or treated with 25 μg/cm^2^ of SiO_2_ for 4 h (right), secondly exposed to apoptotic Jurkat cells for 90 min and then stained by HES. Black arrows and stars indicate the localization of Jurkat cells inside or outside MDM, respectively. SiO_2_ particles are easily visualized in MDM cytoplasm on the right picture. ***p < 0.001; ns, not significant.

**Figure 2:**
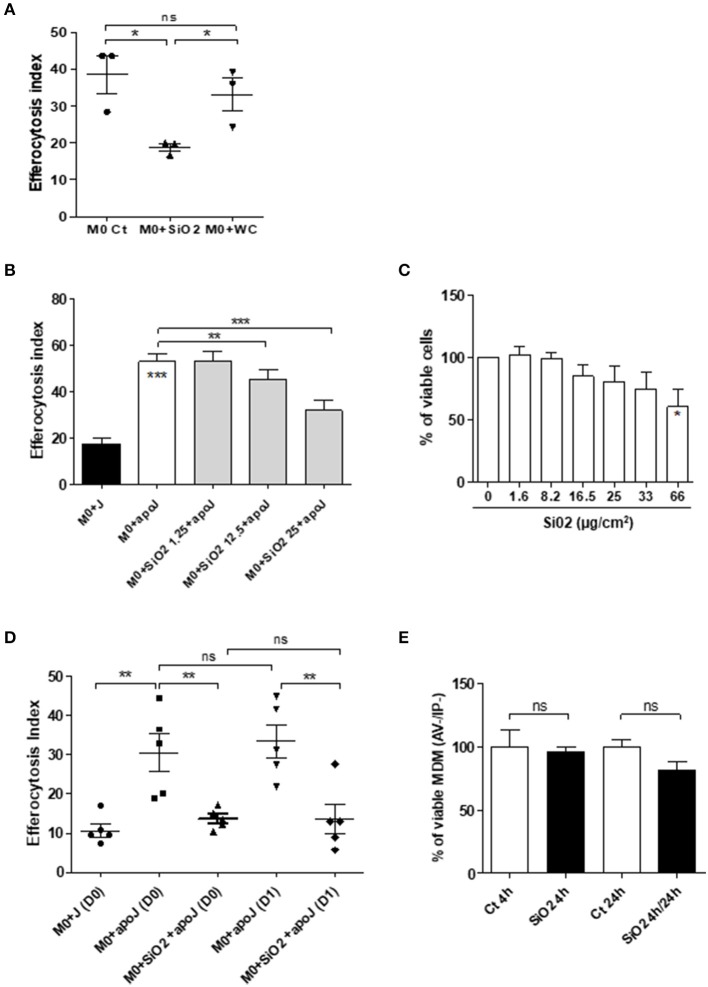
Impaired efferocytosis capacities of silica-exposed MDM *in vitro* is specific, dose dependent, irreversible and is not directly due to the impact of SiO_2_ on cell viability. **(A)** EI of M0 MDM from the same healthy donors, untreated or treated by 25 μg/cm^2^ of SiO_2_ or WC for 4 h and then exposed to apoptotic Jurkat (apoJ) or live Jurkat (J) cells (Experiment on MDM from 3 different healthy donors). **(B)** EI of M0 MDM from the same healthy donors, untreated or treated by the indicated concentrations of SiO_2_ for 4 h and then exposed to apoptotic Jurkat (apoJ) (Experiment on MDM from 3 different healthy donors). **(C)** Determination of MDM cell viability by a WST-1 assay; data expressed in percentage of viable cells (Experiment on MDM from 4 different healthy donors). **(D)** EI of M0 MDM untreated or treated with 25 μg/cm^2^ of SiO_2_ for 4 h, washed and then exposed to apoptotic Jurkat (apoJ) or live Jurkat (J) cells for 90 min the same day (D0) or 24 h later (D1) (Experiment on MDM from 5 different healthy donors). **(E)** Determination, by flow cytometry, of MDM cell viability by AV/IP staining after exposure to 25 μg/cm^2^ of SiO_2_ for 4 h only or, 4 h followed by a 24 h period without SiO_2_ (Experiment on MDM from 4 different healthy donors). *p < 0.05; **p < 0.01; ***p < 0.001; ns, not significant.

**Figure 3:**
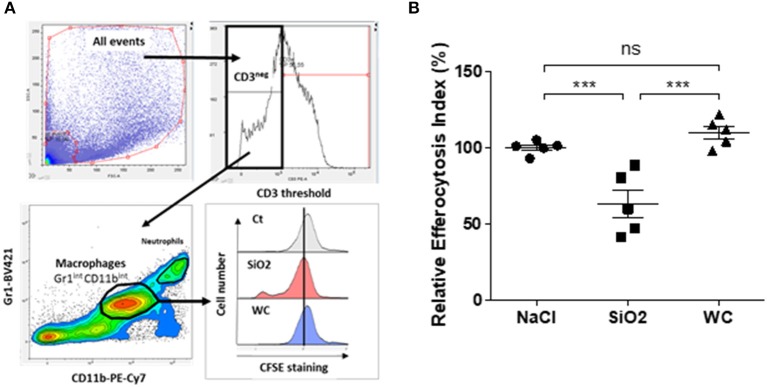
Impaired efferocytosis capacities of alveolar MΦ from mice exposed *in vivo* to SiO_2_. **(A)** Gating strategy used to evaluate efferocytosis capacities of alveolar MΦ from mice. EI of BAL cells obtained from mice exposed to NaCl or to 1.5 mg of SiO_2_ or WC for 4 days and then exposed *in vivo* to CFSE^pos^ apoptotic Jurkat (apoJ) cells, were analyzed by flow cytometry. **(B)** EI of alveolar MΦ which have engulfed apoptotic CFSE^pos^ Jurkat cells (CD3^neg^ Gr1^int^ CD11b^int^ CFSE^pos^ cells). Efferocytosis of mice treated with NaCl as control was set as 100% (*n* = 5 mice in each group). ***p < 0.001; ns, not significant.

MDM exposed to 25 μg/cm^2^ of SiO_2_ were less able to engulf apoptotic Jurkat cells (M0 SiO_2_ + apoJ) than untreated MDM (M0 + apoJ), as the EI of SiO_2_-exposed MDM was significantly lower (17.6 ± 1.7) than the EI of untreated MDM (32.4 ± 2.2) (Figure 1A). This decrease of apoptotic Jurkat cells engulfment and the phagocytosis of SiO_2_ by human MDM was illustrated on Figure 1B (right) when compared to untreated MDM (Figure 1B, left). The EI of human MDM exposed to apoptotic Jurkat cells (M0 + apoJ) was significantly higher (32.4 ± 2.2) than the EI of MDM exposed to non-apoptotic Jurkat cells (M0 + J) (11.1 ± 1.1), confirming the specificity of this efferocytosis assay (Figure 1A). The decrease of efferocytosis in SiO_2_-exposed MDM was specific, as human MDM exposed to the same concentration of tungsten carbide (WC) particles had preserved efferocytosis capacities (38.6 ± 5.0 in M0 Ct + apoJ vs. 33.2 ± 4.5 in M0 + WC + apoJ) (Figure 2A). This impairment of efferocytosis by SiO_2_ in human MDM was dose-dependent (Figure 2B). No toxicity of a 4 h-exposure to various SiO_2_ concentrations (from 1.65 to 33 μg/cm^2^) was observed using a WST1 cell viability assay (Figure 2C), thus suggesting that the impact of 25 μg/cm^2^ of SiO_2_ on efferocytosis was not the consequence of a decreased cell viability in our model of MDM. Moreover, MDM could not retrieve their efferocytosis capacities 24 h (D1) after a 4 h-exposure to SiO_2_ (D0) (Figure 2D) and this effect was not due to an impact of SiO_2_ on cell viability (Figure 2E). In accordance with these findings in human MDM, the relative EI of alveolar MΦ (CD3^neg^ Gr1^Int^ CD11b^Int^ CFSE^pos^) as defined in the gating strategy (Figure 3A) from mice exposed *in vivo* to CFSE^pos^ apoptotic Jurkat cells 4 days after inhalation of SiO_2_ (63.2 ± 9.2), was significantly lower than after inhalation of NaCl as control (100 ± 2.0) or WC particles (110.1 ± 4.1) (Figure 3B).

### SiO_2_-Reduced Phagocytosis of Apoptotic Jurkat Cells Requires SR-B1

**Figure 4:**
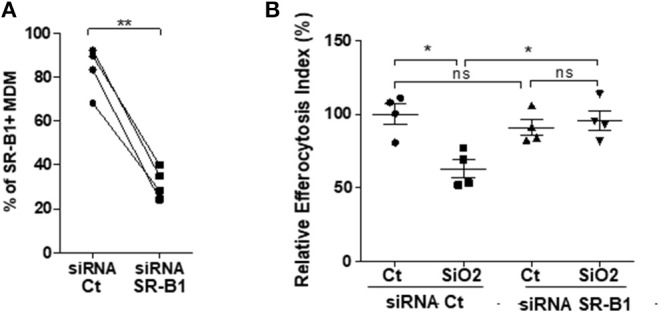
Impairment of efferocytosis by silica in MDM requires SR-B1 expression. M0 MDM from the same healthy donors, were transfected with siRNA Ct or siRNA for SR-B1 for 24 h. Percentages of SR-B1 positive MDM **(A)** and EI of MDM from the same 4 healthy donors **(B)**, untreated or treated to SiO_2_ and then exposed to apoptotic Jurkat (apoJ) or live Jurkat (J) cells for 90 min, were both determined by flow cytometry (Experiment on MDM from 4 different healthy donors). *p < 0.05; **p < 0.01; ns, not significant.

Because SR-B1 has been demonstrated as a SiO_2_ membrane receptor of MΦ (11), we further determined the role of this receptor on the modulation of efferocytosis capacities in SiO_2_-exposed human MDM. A transient transfection of RNAi against SR-B1 significantly reduced endogenous SR-B1 expression in MDM in comparison with cells transfected with a non-targeting siRNA (Ct) (Figure 4A). SiO_2_ significantly reduced the EI in siRNA Ct-transfected MDM but not in siRNA SR-B1-transfected MDM (Figure 4B), demonstrating that SiO_2_-induced reduction of efferocytosis in human MDM is SR-B1 dependent.

### Effects of SiO_2_ on MDM Polarization Markers and on the Expression of Membrane Receptors Involved in Efferocytosis

**Figure 5:**
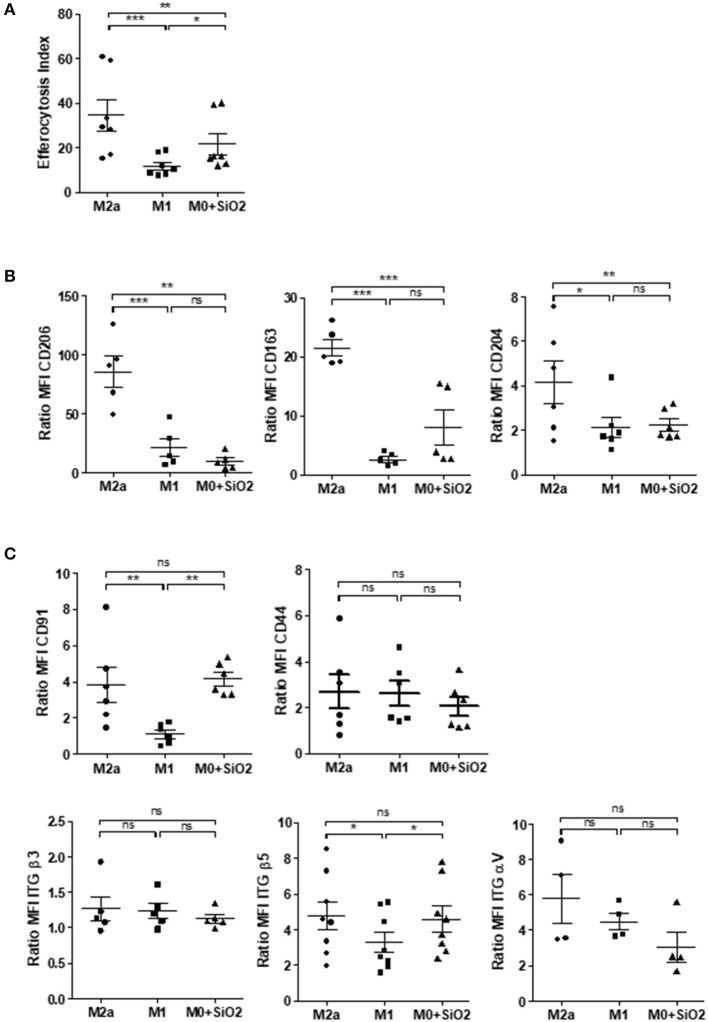
Silica-exposed MDM have a M1-like phenotype. **(A)** EI of M0 MDM from the same healthy donors treated by 25 μg/cm^2^ of SiO_2_ for 4 h or of MDM polarized for 24 h into M2a or M1 and then exposed to apoptotic Jurkat for 90 min (Experiment on MDM from 7 different healthy donors). **(B)** Membrane expressions of CD206, CD163, and CD204 or **(C)** of CD91, CD44, ITGβ5, ITGβ3, and ITGαV in M0 MDM treated by 25 μg/cm^2^ of SiO_2_ for 4 h or in MDM polarized for 24 h into M2a or M1 were determined by flow cytometry; data are expressed as ratio of MFI (Experiment on MDM from 4 to 8 different healthy donors). *p < 0.05; **p < 0.01; ***p < 0.001; ns, not significant.

We secondly explored the possible mechanisms involved in this SiO_2_-related impairment of efferocytosis in human MDM. Because pro-inflammatory M1 MΦ exhibit impaired efferocytosis capacities (21, 27), we first compared the EI of polarized MDM with SiO_2_-exposed MDM. Such as M1 MDM, SiO_2_-exposed MDM had a significant lower EI than M2a MDM (Figure 5A). Three markers down-regulated in M1 MDM (CD206, CD163, and CD204) were also significantly reduced in SiO_2_-exposed MDM when compared to M2a MDM, whereas their expression was similar in SiO_2_-exposed MDM and M1 MDM (Figure 5B). Moreover, SiO_2_ increased the secretion levels of pro-inflammatory M1-related cytokines IL-6, IL-8, and TNFα but not those of CCL18, a typical M2a marker. By contrast, WC had no effect on the secretions of these cytokines (Figure S1). Altogether these results suggest that SiO_2_ modulates the polarization state toward a M1 like-phenotype.

Several membrane receptors are involved in the recognition of apoptotic bodies by MΦ (28) and a decrease of their expression has been involved in the impairment of efferocytosis in M1 MDM (21). Therefore, we evaluated the impact of SiO_2_ exposure on the expression of some of these membrane receptors and we compared their expression with M1 and M2a MDM. Membrane expression of CD91 and ITGβ5 were significantly reduced in M1 MDM when compared to M2a or SiO_2_-exposed MDM (Figure 5C). The expressions of CD91, ITGβ5, CD44, ITGβ3, and ITGαV were similar in M2a MDM and in SiO_2_-treated MDM (Figure 5C). These results suggest that the effect of SiO_2_ on efferocytosis is not due to a SiO_2_ mediated down-expression of these efferocytosis receptors.

### SiO_2_ Induces RhoA/ROCK Pathway in Human MDM

**Figure 6:**
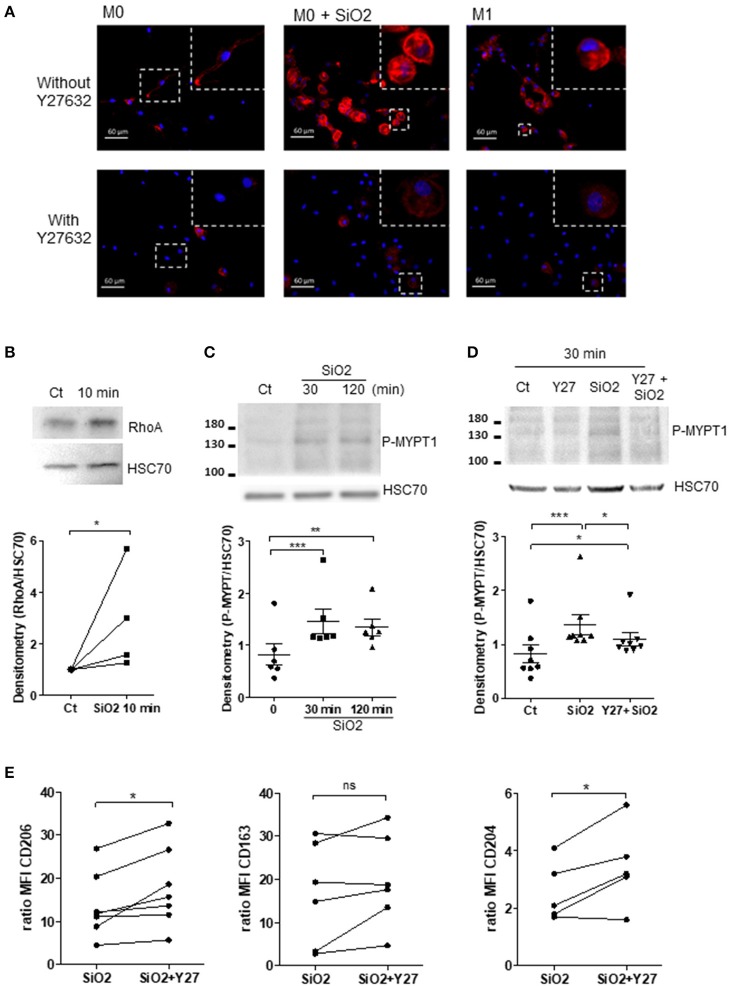
Silica exposure induces cytoskeleton remodeling and alteration of MΦ polarization through activation of the RhoA/ROCK pathway. **(A)** Representative pictures of fluorescence microscopy: F-actin and nuclei were stained by Alexa Fluor 568-phalloidin (red) and DAPI (blue), respectively. M0-MDM were untreated or treated with 25 μg/cm^2^ of SiO_2_ for 10 min or polarized into M1 cells for 24 h. MDM were also pre-treated or not 1 h with the ROCK inhibitor Y27632 at 20 μM. **(B–D)** M0-MDM (Ct) from the same healthy donors were pre-treated or not with 20 μM Y27632 and then untreated or not with 25 μg/cm^2^ of SiO_2_ for the indicated time. The GTP-binding fraction of RhoA was pulled-down as described in Materials and Methods. **(C,D)** Western-blot analyzes of Phospho-MYPT1 expression were performed on whole-cell lysates. The relative levels of the proteins were determined by densitometry (Experiment on MDM from 4 to 8 different healthy donors). **(E)** Effect of Y27632 on the membrane expression of CD206, CD163, and CD204. MDM from the same healthy donors were pre-treated or not 1 h with the ROCK inhibitor Y27632 at 20 μM before exposure to 25 μg/cm^2^ of SiO_2_ for 4 h; data determined by flow cytometry are expressed as ratio of MFI (Experiment on MDM from 5 to 7 different healthy donors). *p < 0.05; **p < 0.01; ***p < 0.001; ns, not significant.

Considering that the RhoA/ROCK pathway modulates cytoskeleton in MDM (29) and that RhoA/ROCK inhibitors may shift M1 into M2 MΦ (30–32) and could restore efferocytosis reduced by chemical pollutants (33, 34), we explored the role of the RhoA/ROCK pathway in the impairment of efferocytosis induced by SiO_2_ exposure. Firstly, a 10 min- SiO_2_ exposure as well as M1 polarization, induced a higher staining for F-actin in comparison with untreated M0 suggesting a cytoskeletal rearrangement (Figure 6A and Figure S2). Pre-treatment by Y27632, a ROCK inhibitor, suppressed F-actin staining both in M1 polarized MDM and SiO_2_-exposed MDM, suggesting that cytoskeletal rearrangements observed in M1 and in SiO_2_-exposed MDM may involve an activation of RhoA/ROCK (Figure 6A and Figure S2). Secondly, SiO_2_ exposure significantly increased RhoA expression in MDM (Figure 6B). The phosphorylation of myosin binding subunit of myosin phosphatase (MYPT), a known target of RhoA/ROCK, increased significantly from 30 min of SiO_2_ exposure (Figure 6C), and this effect was inhibited in the presence of Y27632 (Figure 6D). Thirdly, we observed that, in the presence of Y27632, the membrane expressions of CD206 and CD204 were significantly restored in MDM exposed to SiO_2_. Nonetheless, the expression of CD163 remained unchanged (Figure 6E). Altogether, these data indicate that SiO_2_ activates RhoA/ROCK and that inhibition of this pathway may, at least in part, prevent the loss of M2a polarized markers in SiO_2_-exposed MDM. To support these results on RhoA/ROCK and efferocytosis we also explored EI of GM-CSF derived MDM. Indeed, GM-CSF can activate RhoA/ROCK (35) and GM-CSF MDM are considered as another model of M1 MDM (26, 36). We firstly demonstrated that GM-MDM (GM-M0) had decreased efferocytosis capacities in comparison with M-M0 (Figure S3A). We also demonstrated that GM-M0 had a significantly decreased membrane expressions of CD204 and CD163, therefore confirming their M1-like phenotype (Figure S3B). We lastly confirmed that GM-M0 had a significant higher staining for F-actin (Figure S3C) supporting a GM-CSF mediated activation of RhoA/ROCK, as previously described (35). Altogether, our data support the crucial role of cytoskeleton in the control of MΦ polarization (37).

### Rock Inhibition Partially Restores Impaired Efferocytosis in SiO_2_-Exposed MDM and in MDM From SSc Patients

**Figure 7:**
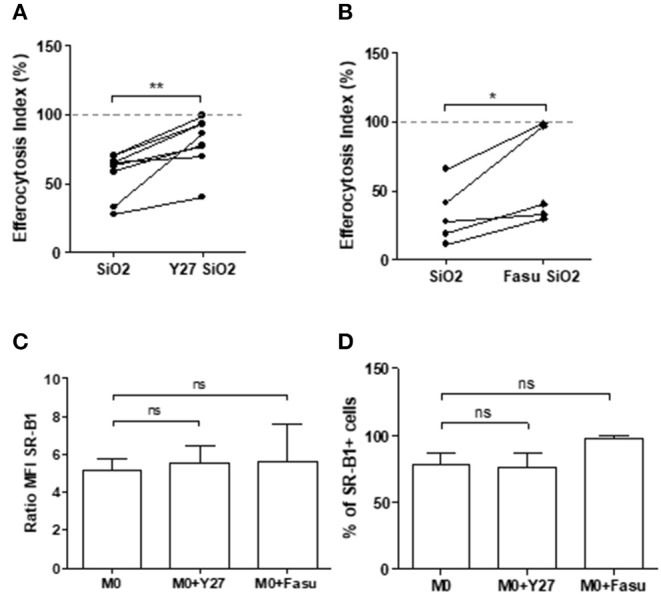
RhoA/ROCK pathway inhibition enhances efferocytosis capacities of silica-exposed MDM and this effect is not due to a down expression of SR-B1 after RhoA/ROCK inhibition. **(A,B)** EI of MDM pre-treated or not with the ROCK inhibitors **(A)** Y27632 or **(B)** fasudil at 20 μM and treated with 25 μg/cm^2^ of SiO_2_ for 4 h and then exposed to apoptotic Jurkat cells for 90 min (Experiment on MDM from at least 5 different healthy donors). **(C,D)** Expression of SR-B1 expressed as **(C)** ratio of MFI and **(D)** percentage of positive cells of MDM treated or not with the ROCK inhibitor Y27632 and Fasudil at 20 μM for 4 h analyzed by flow cytometry (Experiment on MDM from 2 to 5 different healthy donors). *p < 0.05; **p < 0.01; ns, not significant.

**Figure 8:**
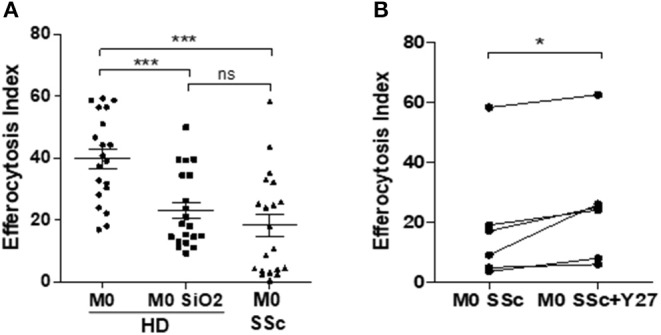
RhoA/ROCK pathway inhibition by Y27632 enhances efferocytosis capacities of MDM from patients with systemic sclerosis. **(A)** EI of M0 MDM from healthy donors untreated or treated with 25 μg/cm^2^ of SiO_2_ for 4 h and of M0 MDM from SSc patients (Experiment on MDM from 20 different healthy donors and 20 different SSc patients). **(B)** EI of M0 MDM from SSc patients pre-treated or not with 20 μM Y27632 before exposure to apoptotic Jurkat cells (apoJ) for 90 min (Experiment on MDM from 6 different healthy donors). *p < 0.05; **p < 0.01; ***p < 0.001; ns, not significant.

The EI of SiO_2_-exposed MDM in the presence of RhoA/ROCK inhibitors such as Y27632 (Figure 7A) or Fasudil (Figure 7B) were significantly higher than the EI of SiO_2_-exposed MDM without these inhibitors. This improvement of efferocytosis capacities by Y27632 or Fasudil in SiO_2_-exposed MDM was not explained by a variation of SR-B1 expression (Figures 7C,D), suggesting that the effect of these two inhibitors was not related to a modulation of the interaction of SiO_2_ with SR-B1, but was more likely due to its direct impact on the RhoA/ROCK pathway. Figure 8A shows that the EI of MDM from patients with SSc, an autoimmune fibrotic disease associated with SiO_2_ exposure, was decreased in comparison with the EI of MDM from HD. This impairment of efferocytosis in SSc MDM was similar to the decrease of efferocytosis induced by an *in vitro* exposure to SiO_2_ in HD MDM. Moreover, EI of SSc MDM was partially restored after treatment by Y27632 (Figure 8B), suggesting that inhibiting RhoA/ROCK pathway may also improve efferocytosis in SSc.

## Discussion

**Figure 9:**
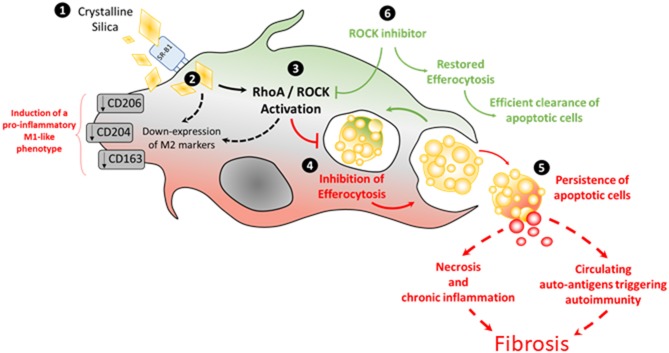
Pathways involved in silica-impaired efferocytosis abilities of MDM [adapted from Lescoat et al. (38), license number: 4681481154582].

This is the first study evaluating the direct effects of SiO_2_ on the efferocytosis capacities of human and mouse MΦ. We demonstrate in this work that SiO_2_ impairs capacities of human MDM and of mouse alveolar MΦ to clear apoptotic Jurkat cells. In human MDM, this effect of SiO_2_ is dose-dependent and requires the scavenger receptor SR-B1. This reduced efferocytosis after SiO_2_ exposure is associated with an induction of a pro-inflammatory M1-like phenotype in human MDM. Moreover, SiO_2_ activates the RhoA/ROCK pathway and ROCK inhibition partly restores efferocytosis capacities of SiO_2_-exposed MDM, demonstrating that RhoA/ROCK pathway is involved in this SiO_2_ induced-impairment of efferocytosis (Figure 6). A reduced efferocytosis may lead to persistent apoptotic cells that may undergo subsequent necrosis leading to a pro-inflammatory environment with delayed resolution of inflammation (17) and fibrosis (Figure 9). Moreover, this persistence of apoptotic cells due to SiO_2_-impaired efferocytosis may concur to the release of intranuclear components that could activate innate and adaptive immunity, triggering or exacerbating SiO_2_-associated systemic autoimmune diseases such as SSc and SLE. In accordance with this hypothesis, MDM from SSc patients have reduced efferocytosis capacities and a treatment by the ROCK inhibitor Y27632 partly restores this defective efferocytosis, confirming the involvement of RhoA/ROCK in this SiO_2_-associated fibrotic disorder.

The link between fibrosis and a defective efferocytosis is strengthened by the existence of a similar impairment of efferocytosis in IPF (22). Moreover, instillation of an excess of apoptotic cells, followed by active efferocytosis, in the early phase of the bleomycin mouse model of pulmonary fibrosis is associated with a less severe progression of the disease in comparison of saline-instilled mice, also suggesting that an effective efferocytosis in the early stage of the disease is protective from subsequent fibrosis (39). Therefore, inhalation of SiO_2_ could inversely result in an intense pulmonary inflammation associated with a defect of efferocytosis leading to a delayed or impaired resolution of inflammation and to fibroblast activation resulting in fibrosis. Impaired efferocytosis by alveolar MΦ would therefore constitutes a key player in the pathogenesis of SiO_2_-induced fibrosis but the direct link between such efferocytosis defect and fibrosis is still to be demonstrated especially *in vivo*. In our study, exposure to SiO_2_ reduced the EI of mouse alveolar MΦ and human MDM in a similar intensity (1.6- and 1.8-fold, respectively). However, we also observed some variation in SiO_2_ effects on EI in some mice; as it is inherent to *in vivo* experiments, the variability of such effects could be partly due to the variable efficiency of the transoral instillation of the particles. The concentration of SiO_2_ in human MDM (25 μg/cm^2^) was similar to previous studies evaluating the impact of SiO_2_ on alveolar MΦ (40). The concentration of SiO_2_ used *in vivo* (1.5 mg/mice), although lower than the concentrations used in mouse models of silicosis (2.5 to 10 mg/mice) (41–43), was able to induce granuloma formation (data not shown). We can thus hypothesize that the observed defect of efferocytosis at 1.5 mg/mice may also occur with higher concentrations of SiO_2_. Other airborne contaminants, such as cigarette smoke, associated with inflammatory lung diseases can also impair efferocytosis (34, 44). Nonetheless, SiO_2_ may have a more significant impact *in vivo*, since, as a mineral particle, it remains persistent in lung tissues and in mediastinal lymphadenopathies of silica-exposed workers many years after exposure cessation (1). In our work, the effect of SiO_2_ on efferocytosis persisted 24 h after discontinuation of SiO_2_ exposure in human MDM, suggesting that the impact of SiO_2_ on the clearance of apoptotic cells is long-lasting. The phagocytic capacity of dendritic cells, also considered as professional efferocytosis cells, was decreased after a direct exposure to SiO_2_ at a concentration similar to the concentration we used in our study, suggesting that silica may also probably alter efferocytosis abilities of these myeloid cells (45). Interestingly, among particles, this effect of SiO_2_ on efferocytosis appeared specific, as another crystalline dust, WC particles, had no significant impact on efferocytosis. WC does not induce pulmonary inflammation or fibrosis after tracheal instillation in mice models (41) and is therefore a crystalline dust considered as non-toxic, especially for MΦ (46). Altogether, these data suggest that it is the nature of the particles rather than their accumulation in MΦ that participates in the disruption of efferocytosis.

The limitations of this study include the use of only one type of crystalline silica; indeed, other types of crystalline silica may have different effects than DQ12. Nonetheless, DQ12 is largely used in the literature *in vitro* and *in vivo* to assess silica hazards (41). Moreover, as the exposure to amorphous silica is not associated with autoimmune diseases, it could be interesting to evaluate its effects on efferocytosis to confirm that it does not impair this function, contrarily to crystalline silica. The incomplete expression analysis of the efferocytosis receptors is also another limitation of this study. Indeed, since the role of efferocytosis is crucial for tissue homeostasis, there are numerous types of such receptors at the cell surface and, an extent analysis of SiO_2_ impact on all of them was beyond the scope of this study.

Considering the issue of MΦ polarization in CTDs, our results are concordant with previous studies. Indeed, in our work, SiO_2_ affected MΦ phenotype and shifted them into MΦ sharing some phenotypic characteristics of M1 MΦ as suggest by the down-expression of the M2 membrane markers CD206, CD204, and CD163 and by the upregulation of pro-inflammatory cytokines. Blood MDM in SLE are characterized by a similar down-regulation of CD206 (47). As SSc is both a fibrotic and inflammatory disorder, the polarization profile is more complex, although blood MDM are also characterized by some phenotypic features associated with M1 polarization such as a down-expression of CD204 (12). On the contrary, in fibrotic tissues such as lungs and skin in SSc, tissue MΦ express M2 markers such as CD163. It has been recently highlighted that the patients with SSc had higher blood levels of silica particle in comparison healthy controls (48). We hypothesize that for some patients this circulating silica could participate both to the alteration of polarization profile in SSc-MDM and to the impairment of their efferocytosis capacities.

The activation of RhoA/ROCK by SiO_2_ is concordant with the involvement of this pathway in autoimmune disorders classically associated with silica exposure in human and characterized by a defective efferocytosis with production of antinuclear antibodies (ANA). Indeed, in mouse models of SLE, ROCK1/2 are spontaneously up-regulated and Y27632 significantly reduces serum levels of pro-inflammatory cytokines. Efferocytosis and activation of RhoA/ROCK could represent a key pathogenic process at the crossroads of systemic autoimmunity, MΦ and silica exposure: in mouse models of SLE, SiO_2_ inhalation is associated with more severe visceral manifestations of the disease and higher levels of ANA (43, 49, 50). ANA produced after SiO_2_ exposure specifically target apoptotic cells (51) and an altered efferocytosis after SiO_2_ inhalation could lead to an excess of uncleared apoptotic cells with subsequent increased production of these ANA. Consistently, in a mouse model of SSc, fasudil, another ROCK inhibitor, significantly reduces lung inflammation, fibrosis and also serum levels of anti-DNA-topoisomerase-1 ANA (24). The links between a possible restoration of efferocytosis and this reduction of autoimmune features and/or fibrotic manifestations by ROCK inhibitors in SLE and SSc have never been established to date. Interestingly, significant associations between ROCK1/2 and RhoA gene polymorphisms and SSc have been reported, which also strengthens the possible role of this pathway in this systemic autoimmune disorder (52). New Rho inhibitors have been recently design for the treatment of SSc with promising results on dermal fibrosis in the bleomycin SSc mouse model (23). Beyond fibroblasts, the authors announce that identifying other biological targets of these new Rho inhibitors is a new key step for scleroderma research (23). Our results suggest that MΦ could constitute such relevant targets. Concerning the direct link between autoimmunity and fibrosis, defective efferocytosis might contribute to an excess of autoantigens with subsequent higher amount of immune-complexes (IC) and, recently, a direct activation of scleroderma fibroblasts by IC has been described [(53); Figure 9], strengthening the hypothesis that enhancing efferocytosis to reduce circulating IC could be a relevant strategy to limit fibrosis, at least in the early phase of the disease and/or in patients with uncontrolled inflammation. In our work, MDM from SSc patients had impaired efferocytosis capacities and this impairment was similar to the defective efferocytosis capacities of *in vitro* SiO_2_-treated MDM from HD. Treatment by Y27632 11enhanced efferocytosis in SSc MDM, suggesting that an activation of ROCK may be involved in the reduced clearance of apoptotic cells by SSc MDM. Nonetheless, the relevance and the clinical impact of such an enhancement of efferocytosis in SSc patients is still to be determined. Although, the direct links between SiO_2_ exposure and ROCK activation in SSc cannot be established in our study, more than 25% of SSc patients from our cohort have a history of silica exposure (54). Nonetheless, a detailed evaluation of the history of silica exposure, with precise dating of the time and duration of exposure and their correlation with impaired efferocytosis would be necessary to evaluate if this impairment could be considered as a biomarker of silica exposure and silica hazards in our SSc patients. Moreover, the impact of SiO_2_ exposure and the exploration of efferocytosis in mouse models of CTDs in future studies may help to better characterize the interactions between SiO_2_, RhoA/ROCK and defective clearance of apoptotic cells.

## Data Availability Statement

The datasets generated for this study are available on request to the corresponding author.

## Ethics Statement

The studies involving human participants were reviewed and approved by Committees for protection of persons (CPP) Ouest-V France, CPP approval N°: 2015-A01221-48; study N°.15/26-988. The patients/participants provided their written informed consent to participate in this study. This animal study was reviewed and approved by Committee on the Ethics of Animal Experiments of the French government (agreement of V. Lecureur #17011).

## Author Contributions

AL, AB, and VL conceived and designed experiments. AL, AB, ML, YA, CM, and VL performed experiments. AL, AB, ML, VL, LV, and OF analyzed the data. PJ and SJ contributed reagents and materials. All the authors have contributed to the drafting and writing of the manuscript and added substantial modifications to the manuscript.

### Conflict of Interest

The authors declare that the research was conducted in the absence of any commercial or financial relationships that could be construed as a potential conflict of interest.
