# Supplementary figures and images for: Crystalline Silica Impairs Efferocytosis Abilities of Human and Mouse Macrophages: Implication for Silica-Associated Systemic Sclerosis
Source: Front Immunol. 2020 Feb 18;11:219. doi: 10.3389/fimmu.2020.00219 (PMC7039938; doi:10.3389/fimmu.2020.00219)

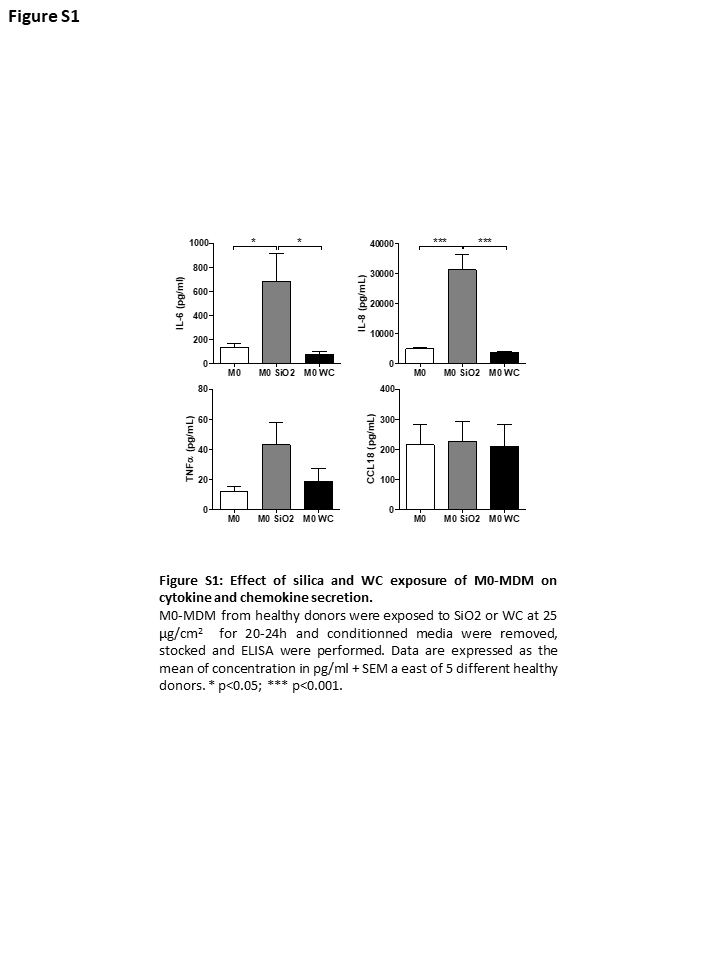

Supplement: Supplementary file 1 [file Image_1.TIF]

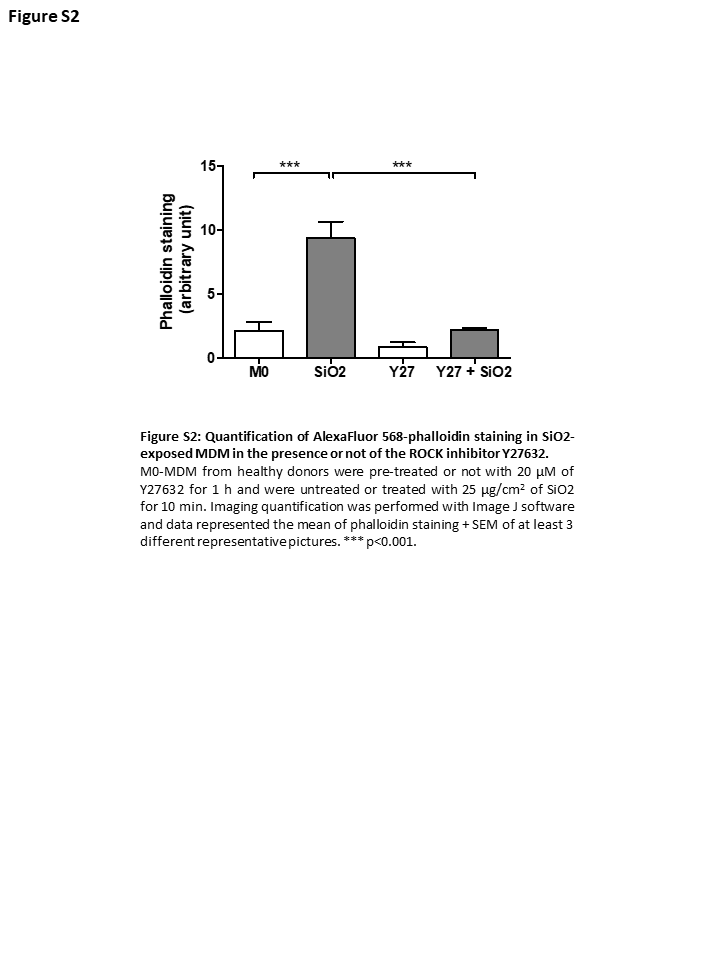

Supplement: Supplementary file 2 [file Image_2.TIF]

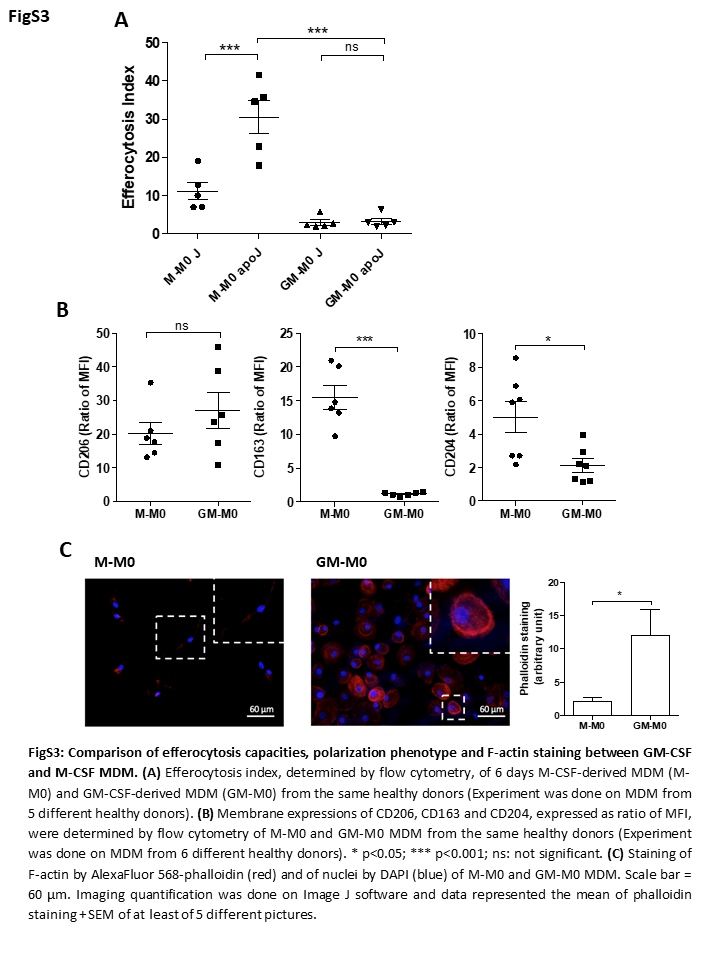

Supplement: Supplementary file 3 [file Image_3.tif]
